# Molecular Events Occurring in Lipophagy and Its Regulation in *Flaviviridae* Infection

**DOI:** 10.3389/fmicb.2021.651952

**Published:** 2021-05-21

**Authors:** Keke Wu, Shuangqi Fan, Linke Zou, Feifan Zhao, Shengming Ma, Jindai Fan, Xiaowen Li, Mingqiu Zhao, Huichao Yan, Jinding Chen

**Affiliations:** ^1^College of Veterinary Medicine, South China Agricultural University, Guangzhou, China; ^2^Guangdong Laboratory for Lingnan Modern Agriculture, Guangzhou, China

**Keywords:** *Flaviviridae*, lipid droplets, lipophagy, HCV, DENV

## Abstract

Diseases caused by *Flaviviridae* have a wide global and economic impact due to high morbidity and mortality. *Flaviviridae* infection usually leads to severe, acute or chronic diseases, such as liver injury and liver cancer resulting from hepatitis C virus (HCV) infection, dengue hemorrhagic fever (DHF) or dengue shock syndrome (DSS) caused by dengue virus (DENV). Given the highly complex pathogenesis of *Flaviviridae* infections, they are still not fully understood at present. Accumulating evidence suggests that host autophagy is disrupted to regulate the life cycle of *Flaviviridae*. Organelle-specific autophagy is able to selectively target different organelles for quality control, which is essential for regulating cellular homeostasis. As an important sub process of autophagy, lipophagy regulates lipid metabolism by targeting lipid droplets (LDs) and is also closely related to the infection of a variety of pathogenic microorganisms. In this review, we briefly understand the LDs interaction relationship with *Flaviviridae* infection, outline the molecular events of how lipophagy occurs and the related research progress on the regulatory mechanisms of lipophagy in *Flaviviridae* infection. Exploring the crosstalk between viral infection and lipophagy induced molecular events may provide new avenues for antiviral therapy.

## Introduction

Viruses, as highly parasitic microorganisms, therefore have to rely entirely on the host cell processes to complete their life cycle due to the lack of essential metabolic machinery required for replication or assembly. The *Flaviviridae* consists of a group of enveloped viruses with positive single strand RNA genomes, including hepatitis C virus (HCV) of the genus *Hepacivirus*, dengue virus (DENV) and Zika virus (ZIKV) of the genus *Flavivirus*, bovine viral diarrhea virus (BVDV) and classical swine fever virus (CSFV) of the genus *Pestivirus* (Wu et al., 2015). Notably, diseases caused by *Flaviviridae* have a wide global and economic impact due to high morbidity and mortality. For instance, DENV causes the world's most prevalent mosquito-borne flavivirus (FLV) disease, placing nearly 3.9 billion people, mainly in poor countries, at constant risk of infection due to the lack of completely effective vaccines and antivirals against DENV. Recently, an increasing number of studies have shown that *Flaviviridae* extensively regulate host lipid metabolism, underscoring the importance of LDs in viral infection (Martín-Acebes et al., 2019; Cloherty et al., 2020; Roberts and Olzmann, 2020).

LDs are now recognized as highly dynamic subcellular organelles that play a central role in lipid metabolism and are connected to diverse cellular processes like protein storage, fatty acid trafficking, cellular signaling, and host defense (Olzmann and Carvalho, 2019). Although LDs have potential defensive effects on *Flaviviridae*, they are broadly manipulated for replication and production of viruses (Filipe and McLauchlan, 2015; Sun et al., 2017).

Autophagy (also known as macroautophagy), as an intracellular degradation system that maintains cellular metabolic homeostasis and cell survival when cells face various stresses such as nutrient deprivation and accumulation of damaged organelles, is an essential defensive cellular program. Autophagy for the degradation of various organelles has been well-known in the last century, but its function in the degradation of LDs has been intensively studied only in recent years (Anding and Baehrecke, 2017). The process by which intracellular lipids are transported to lysosomes for breakdown by autophagosomes, termed “lipophagy,” often referred to simply as lipophagy, provides another potential avenue for regulating intracellular lipid levels (Singh et al., 2009). As a selective autophagic process, lipophagy not only rapidly clears aggregated lipids, but also provides energy for the replication of some pathogenic microorganisms. Thus, in-depth mining of the molecular mechanism of lipophagy and the interaction with *Flaviviridae* infection may provide us with clues and theoretical guidance for further elucidating the infection mechanism of *Flaviviridae* and the development of new antiviral therapeutics.

## Overview of LDs

LDs are endoplasmic reticulum derived organelles that store neutral lipids. Although present in almost all types of cells in different organisms, LDs are highly heterogeneous and dynamic among homogeneous cells, manifesting as different numbers and sizes. Even within the same cell, LDs swell or contract in response to cellular signals (Zhang et al., 2017; Olzmann and Carvalho, 2019). Unlike most other intracellular organelles, LDs are formed by a hydrophobic core mainly composed of triacylglycerol (TG) and cholesteryl esters (CE), which is wrapped with a monolayer of phospholipid molecules (Gao et al., 2019).

### LDs Biogenesis

One of the keys to LDs biogenesis is the synthesis of neutral lipids, in which the synthesis of TG and CE is a prerequisite for LDs formation, affecting the morphology and size of LDs. In mammalian cells, the enzymes responsible for catalyzing the final step in the TG biosynthetic pathway are diacylglycerol acyltransferases 1 and 2 (DGAT1 and DGAT2) catalyze the last step in the TG biosynthesis pathway, while acyl-CoA cholesterol acyltransferases 1 and 2 (ACAT1 and ACAT2) determine CE synthesis (Fujimoto et al., 2008). These enzymes are mainly found in endoplasmic reticulum (ER) membranes, which is also one of the currently direct evidences supporting that LDs originate from the ER (Ohsaki et al., 2014).

### LDs Proteome

Numerous proteins are found on the surface of the lipid droplet including Perilipin and other droplet-associated proteins, e.g., structural proteins, enzymes involved in various aspects of cholesterol and fatty acid metabolism, and proteins that function as regulators of membrane traffic, which are important target molecule for the regulation of LDs structure and function (Roberts and Olzmann, 2020). The PAT domain family of the perilipin A (PLIN A), adipose differentiation related protein (ADRP/PLIN2) and tail interacting protein of 47-kDa (TIP47/PLIN3) were the first protein identified in maintaining the stability of LDs morphology (Sztalryd and Brasaemle, 2017). Lipolytic enzymes such as adipose triglyceride lipase (ATGL), hormone- sensitive lipase (HSL) and neutral cholesterol ester hydrolase (nCEH) are critical for the regulation of intracellular neutral lipid degradation (Zimmermann et al., 2004; Vanni, 2017). Additionally, the Ras-related GTP-binding proteins (Rabs) family, soluble NSF attachment protein receptors (SNAREs) proteins and other LDs trafficking-related proteins play an important role in regulating signal transduction and cellular processes, which include vesicle transport, cytoskeleton formation and membrane trafficking (Roberts and Olzmann, 2020).

## Role of LDs in Viral Infection

Accumulating evidence indicates that LDs, as dynamic complex and multi-biological functional organelles, can interact with various organelles, and their numbers and activity are closely related to the occurrence, development, and prognosis of many diseases (Vanni, 2017; Olzmann and Carvalho, 2019). Various intracellular parasitic pathogens, including viruses, some bacteria, and parasites, specifically utilize host LDs to complete a series of life cycle (Roingeard and Melo, 2017). Pathogenic microorganisms particularly *Flaviviridae* are able to deplete LDs during infection to hijack host lipid metabolism for energy and to promote the proliferation of pathogens by utilizing LDs as a platform for viral replication and assembly (Roingeard and Melo, 2017). In the latest studies, LDs have been found to act as molecular switches in innate immune, thus forming the first line of intracellular defense, and the inflammatory mediators released from LDs, as well as antiviral proteins on their surface, play important roles in defense against microbial infections (Bosch et al., 2020).

### LDs-Involvement in *Flaviviridae* Life Cycle

Within the last few years, several reviews have been published on the utilization of LDs by *Flaviviridae* (Zhang et al., 2017; Martins et al., 2018). Herein, we briefly elaborate the progress of HCV-LDs interaction section (as is shown in Table 1). HCV is an enveloped RNA virus that approximately causes 130–170 million cases of viral hepatitis worldwide. Steatosis, a hallmark of HCV infection, occurs in 50–70% of chronically infected individuals. Given that LDs are important components of hepatic lipid stores and serve as platforms for HCV particle aggregation, this dynamic subcellular organelle plays a gatekeeper role in the pathogenesis of viral hepatitis (Filipe and McLauchlan, 2015). The HCV genome encodes 10 proteins-consisting of the structural proteins, core, E1 glycoprotein and E2; and the non-structural viral proteins p7, NS2, NS3, NS4A, NS4B, NS5A, and NS5B (Lambert et al., 2014). Most of these non-structural proteins are required for RNA replication, such as NS3/4A which has NTPase and helicase activities, NS5B which is an RNA-dependent RNA polymerase, as well as the NS5A which is required for and localizes at the sites of both HCV genome replication and virion assembly (Reiss et al., 2011).

**Table 1:** Summary of *Flaviviridae*-LD interactions.

**Virus**	**Viral factor(s)**	**LD-associated proteins**	**LD-associated proteins function**	**Functional target**	**References**
HCV	NS5A, Core	DGAT1	Catalyzes the final step in the TG biosynthetic pathway and mediates the formation of LDs	Serve as a bridge molecule between the core protein and NS5A to ensure the complete HCV machinery addressed to the same lipid droplet subset	Hourioux et al., 2007; Roingeard and Hourioux, 2008; Herker et al., 2010; Silvas et al., 2020
	NS5A	TIP47/PLIN3	A member of the PAT family, mainly involved in the rapid assembly of nascent LDs	Recruitment of NS5A on LDs to promote the release of HCV particles	Vogt et al., 2013
	NS5A, Core	Rab18	A member of the small Rab GTPase family involved in intracellular vesicular trafficking	Recruitment of replication sites to LDs	Salloum et al., 2013
	NS5A	ApoE	Apoli-poprotein E	The C-terminal alpha-helix domain of ApoE interacts with NS5A and determines assembly of infectious HCV particles	Benga et al., 2010; Cun et al., 2010
		Viperin	An interferon inducible antiviral protein located on the surface of LDs	Interact with HCV NS5A through its C-terminal region to inhibit viral replication	Wang et al., 2012; Ghosh et al., 2020
	Unknow	PLIN2	A member of the PAT family, also known as adipose differentiation-related protein, ADRP or adipophilin, ADFP	Trafficking the core and NS5A to LDs, and for formation of functional low-density HCV particles prior to ApoE incorporation	Lassen et al., 2019
DENV	NS3	Rab18	A member of the small Rab GTPase family involved in intracellular vesicular trafficking	Recruit the enzyme fatty acid synthase to sites of DENV replication and to interact with DENV NS3 protein to promote fatty acid biosynthesis	Tang et al., 2014
	C	TIP47/PLIN3	A member of the PAT family, mainly involved in the rapid assembly of nascent LDs	The interaction is dependent of the high intracellular concentration of potassium ions	Carvalho et al., 2012
	NS4A; NS4B	AUP1	Ancient ubiquitous protein 1, lipid droplet accumulation and endoplasmic reticulum (ER) protein quality control; modulate the onset of lipophagy	Trigger lipophagy and drive virus production	Zhang J. et al., 2018
BVDV	NS5A	Unknow		Localize to LDs	Isken et al., 2014
CSFV	NS5A	Rab18	A member of the small Rab GTPase family involved in intracellular vesicular trafficking	Mediate viral replication and assembly in swine umbilical vein endothelial cells	Zhang et al., 2020
		Rab1A	A member of the small Rab GTPase family involved in the regulation of endoplasmic reticulum to Golgi vesicle trafficking	Be required for assembly of CSFV particle	Lin et al., 2018
		Viperin	An antiviral protein induced by interferons	Inhibit CSFV replication by binding to the CSFV NS5A protein	Xu et al., 2019
	NS4B	Rab5	A member of the small Rab GTPase family; Key regulators of membrane component trafficking	Enhance CSFV proliferation; Facilitate formation of NS4B related complex	Lin et al., 2017

HCV is one of the most extensively studied pathogens that interacts with LDs. Studies have shown that HCV virion can be tightly associated with lipoproteins to form a hybrid particle that has been called lipoviroparticle (LVP) (Reiss et al., 2011; Boyer et al., 2014). In addition, substantial accumulation of LDs was found in HCV-infected cells *in vitro*, and both core and the non-structural protein NS5A could localize to LDs (Barba et al., 1997; Reiss et al., 2011). HCV core protein interacts with DGAT1 in the ER membrane, and this interaction activates DGAT1 and promotes TG synthesis, which is required for the trafficking of core protein to LDs (Hourioux et al., 2007; Roingeard and Hourioux, 2008; Herker et al., 2010). Unlike the nearly exclusive localization of the core protein to LDs, HCV NS5A simultaneously localizes to the ER. It has been noted that HCV NS5A similarly interacts with DGAT1, which may serve as a bridge molecule between the core protein and NS5A to ensure that the complete HCV machinery is addressed to the same lipid droplet subset (Camus et al., 2013). Extensive research has shown that NS5A can also interact with lipid droplet associated proteins TIP47 and Rab18 to promote virion formation (Ploen et al., 2013; Salloum et al., 2013; Vogt et al., 2013). In addition, NS5A with RNA binding properties can transport viral RNA from the replication site to LDs to interact with core protein thereby promoting viral RNA encapsidation, nucleocapsid formation, and virus assembly (Ferraris et al., 2013). However, inhibition of either binding of viral proteins to lipid droplet proteins or the LDs generation results in reduced viral replication capacity (Miyanari et al., 2007). In agreement with this idea, the silencing of Rab18, a lipid droplet-associated cellular protein which binds to NS5A, reduces the association of other HCV replication complex components with LDs (Salloum et al., 2013). In addition, in a recent study, a defect in PLIN2/ADRP, the major LDs coat protein in hepatocytes, restricted the LD access for HCV core and NS5A proteins and reduced intracellular infectious HCV particle formation (Lassen et al., 2019).

Similarly, DENV infections could also induce the accumulation of LDs, and the mature capsid protein localized on the surface of LDs is responsible for virion assemble (Samsa et al., 2009; Carvalho et al., 2012; Randall, 2018). Besides, DENV NS3 protein interacts with Rab18, which recruits the enzyme fatty acid synthase to sites of DENV replication (Tang et al., 2014). For instance, BVDV NS5A protein similarly localizes to LDs, but it is not known to which LDs-associated protein it specifically binds (Isken et al., 2014). Recent studies show that the LDs-associated proteins Rab18 and Rab1A interact with CSFV NS5A protein, an essential replicase component, which have been shown to mediate viral replication and assembly process (Lin et al., 2018; Zhang et al., 2020). Additionally, Rab5, which also belongs to the family of small GTPases, enhances CSFV proliferation and interacts with the viral NS4B protein to promote the formation of NS4B associated complexes (Lin et al., 2017). Interestingly, the other viruses can also interact with LDs, such as enterovirus71 (EV71), rotaviruses (RV) of the *Reoviridae* family et al. (Zhu et al., 2015; Lever and Desselberger, 2016).

### LDs-the First Line of Intracellular Defense

In higher eukaryotes, the host has also effectively co-evolved with pathogens in a game process with the pathogen microbe, and the possibility that mammalian LDs has a direct regulatory role in innate immune emerged from recent studies. When host cells are infected by pathogenic microorganisms, LDs not only produce corresponding inflammatory mediators that regulate immune response, but also place a large number of proteins with antibacterial and antiviral properties on LDs and assemble them together to form a complex against pathogen infection (Bosch et al., 2020).

In that study, a comparative mass spectrometric was used to analyze LDs differential-associated proteins in response to LPS, which indicating that the down-regulation of PLIN5 was able to promote physical and functional uncoupling between LDs and mitochondria, reduce fatty acid metabolism and increase the contact of LDs-bacteria (Bosch et al., 2020). In contrast, PLIN2 was the most up-regulated perilipins on LPS-LDs, which helps the accumulation of several interferon-inducible proteins, including Viperin, IGTP, IIGP1, TGTP1, IFI47 et al. In addition, LPS-LDs also accrued a broad-spectrum antimicrobial peptide with chemotactic properties—cathelicidin (CAMP), Which is more resistant to different bacterial species such as *Escherichia coli*, methicillin-resistant *Staphylococcus aureus*, and *Listeria monocytogenes* (Bosch et al., 2020).

Previously, it was shown that N-terminal α-helical structure of Viperin localizes itself on the side of the ER close to the cytoplasm as well as on the surface of LDs. Viperin can also interact with HCV NS5A through the C-terminal region to interfere with the association of NS5A and intracellular lipid raft membranes and inhibit HCV RNA replication, which indicating the importance of LDs association with NS5A during HCV replication (Bosch et al., 2020). Similarly, it was recently shown that Viperin is also able to inhibit CSFV replication by binding to the CSFV NS5A protein (Xu et al., 2019).

Furthermore, although some immune related regulatory mechanisms of LDs in the condition of pathogenic microorganism infection are not fully defined, the function of LDs involved in immune regulation has also received sufficient attention from academia. By revealing the detailed process and specific mechanism of the interaction between LDs and pathogenic microorganisms, targeting LDs as an antiviral strategy can provide new ideas and innovation for the clinical control and treatment of pathogenic microorganism infections in the future.

## Regulation of LDs Metabolism by Lipophagy

### Discovery of Lipophagy

**Figure 1:**
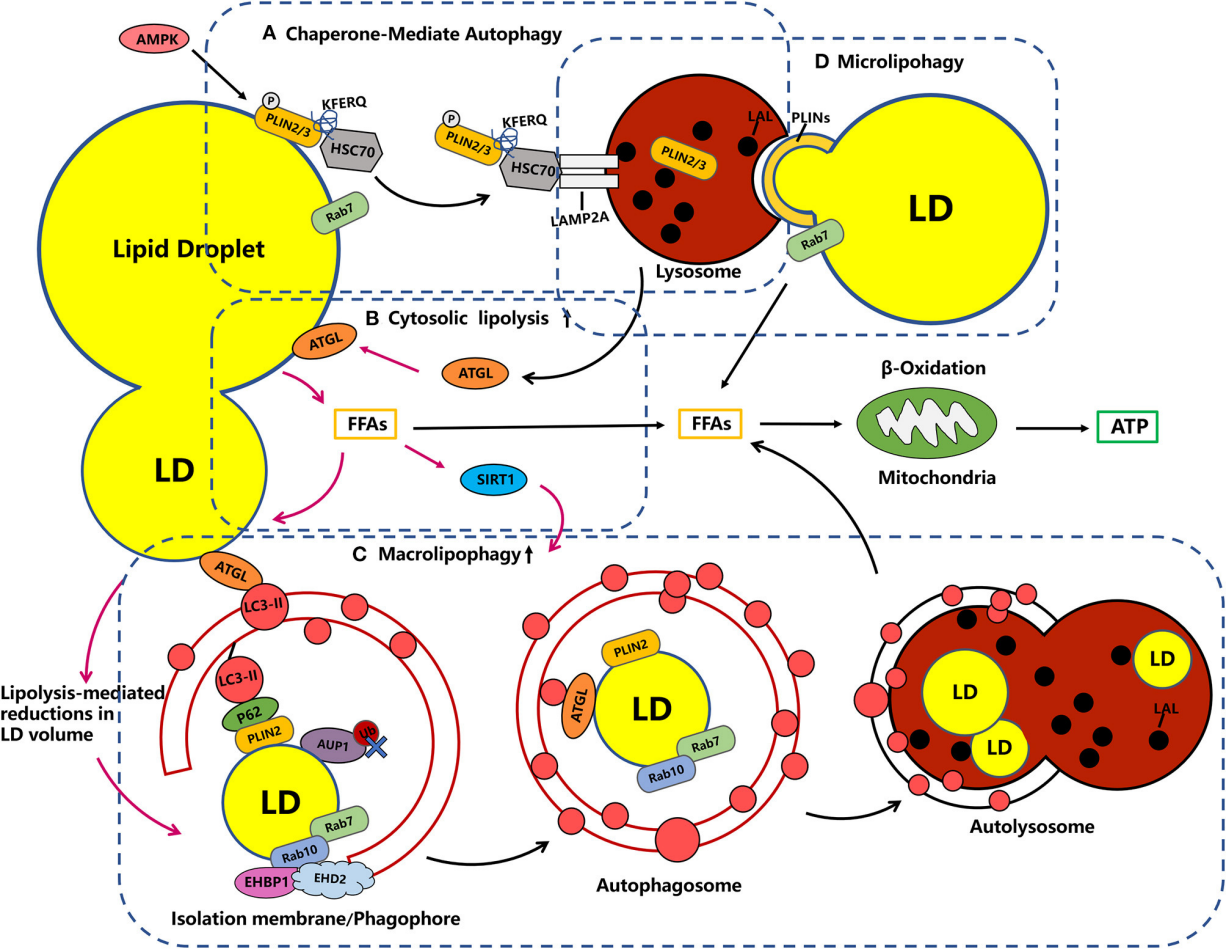
Crosstalk in the metabolic machinery of LDs. **(A)** Chaperone-Mediate Autophagy (CMA). LDs catabolism early degrades PLIN2 through CMA, a process that is important for the recruitment of ATGL and autophagic membranes to LDs. In the CMA, PLIN2/3 proteins, which normally coat the LDs and block access to neutral lipases, are phosphorylated (P) by AMPK and tagged by the adapter HSC70, which promotes LAMP2A-mediated translocation into the lysosome. Rab7 has also been implicated in the formation of LD–lysosome contacts and the transfer of PLIN2 from LDs to lysosomes. **(B)** Cytosolic lipolysis: ATGL targets large LDs upstream of lipophagy, which is restricted to small LDs, and releases fatty acids that can be re esterified and packaged into small LDs. Besides, the ATGL has been shown to be a necessary and sufficient positive regulator of lipophagy induction acting through the deacetylase SIRT1, suggesting tight co-ordination between two lipolytic pathways (note with pink one-way arrow). **(C)** Macrolipophagy: Autophagosomes engulf small LDs or engulf part of large LDs. The recruitment of autophagosome membranes can be mediated LC3-interacting regions (LIR)-containing adapter proteins, such as P62, one possible candidate for a lipophagy receptor, as it associates with LDs by interaction with PLIN2 and is required for lipophagy. Rab7 dependent rab10 and its complex EHBP1-EHD2 are required for the recruitment of LC3-positive autophagic membranes to LDS, which together could drive the extension of the autophagosome membrane around the LDs for engulfment. **(D)** Microlipophagy: microlipophagy reflects the direct and transient interactions of lysosomes with LDs as a means to degrade LD-derived lipids. The LD-lysosome contacts may be relevant for PLINs, while there is currently a lack of evidence demonstrating microlipophagy in mammalian cells.

Recently, increasing investigations into the biological functions of LDs have revealed that LDs are not just inert intracellular storage depots of cellular neutral lipids, but rather a complex and versatile organelle with diverse morphology, which has key roles in lipid metabolism, membrane traffic, protein degradation, and cellular signaling (Henne et al., 2019). The biological function of LDs is to provide ATP to the body by releasing free fatty acids (FFAs) and transporting them to mitochondria for β-oxidation during energy deprivation, fasting, and exercise (Figure 1; Welte and Gould, 2017; Santucci and Canaan, 2018; Dagpo et al., 2020). In mammalian adipocytes, changes in intracellular energy and hormone levels activate lipolysis, which orchestrates TG hydrolysis to generate energy under the action of lipolytic enzymes present in the cytoplasm, such as ATGL, HSL, and monoacylglycerol lipase (MGL) (Welte and Gould, 2017; Santucci and Canaan, 2018). Previous studies have suggested that lipases regulating TG metabolism exist not only in the cytoplasm but also in lysosomes (Czaja and Cuervo, 2009). Subsequent report further tested this notion, a lysosomal acid lipase (LAL) capable of breaking down TG and CE in LDs into free fatty acids and free cholesterol for cellular metabolism (Gomaraschi et al., 2019). Moreover, earlier studies on the regulation of acid lipase activity using the non-specific chemical drugs, ammonium chloride or chloroquine, showed that acid lipase in lysosomes contributes to the degradation of intracellular LDs (Singh et al., 2009).

The process by which intracellular LDs or lipids are degraded by the autophagy lysosomal pathway is also referred to as “lipophagy” and belongs to the category of selective autophagy, which is frequently exploited by *Flaviviridae* to facilitate viral replication (Heaton and Randall, 2010). Lipophagy was first reported in hepatocytes, as lipid stores are maximal within hepatocytes. In that study, the inhibition of autophagy by using 3-methyladenine (3MA) or knockdown of the autophagy related 5 (ATG5), significantly increased the TG content in hepatocytes (Singh et al., 2009; Singh and Cuervo, 2012). Further evidence for the presence of lipophagy in hepatocytes was provided by the finding that LDs in hepatocytes were encapsulated with autophagic vesicles and the autophagosomes colocalized with LDs (Carmona-Gutierrez et al., 2015). The phenomenon of LDs degradation by autophagy was also confirmed in the liver of mice. Precisely, electron microscopy analysis of autophagic vesicles in mouse liver revealed the presence of LDs only, or mixed with other contents (Angelini et al., 2016). Under starvation, FFAs were elevated and fatty acid uptake capacity was enhanced in mice livers, and as starvation deepened, the percentage of lipophagic structures as well as the content of TG and CE in autophagic vesicles increased (Ward et al., 2016). Additional studies have shown that colocalization of autophagy marker proteins with LDs is reduced and that both LDs and content, TG are reduced in autophagy deficient mouse livers (Singh et al., 2009). However, the size of LDs often exceeds that of autophagosomes, therefore, we speculate that autophagosomes mainly engulf small LDs or engulf part of large LDs.

### Molecular Mechanisms Underlying the Occurrence of Lipophagy

#### Cytosolic Lipolysis Synergizes With Lipophagy

With the deepening of research on autophagy and lipid metabolism, researchers have found that selective autophagy, particularly the lipophagy machinery, plays a key regulatory role in the dynamic degradation of LDs components. As mentioned above, lipases are not only found in the cytoplasm, and the cytosolic lipolysis and lipophagy have synergistic functions in LDs catabolism. As shown in Figure 1, in the early catabolism of LDs, PLIN2 is degraded through chaperone mediated autophagy (CMA), which is an important process for the recruitment of ATGL, the cytosolic lipolysis associated lipase, and autophagic membranes to LDs (Figure 1A; Kaushik and Cuervo, 2015). Previous studies have found that cold (4°C) stimulation can induce the interaction between microtubule-associated protein light chain 3 (LC3) and ATGL on the surface of LDs in mice liver. Then, the mutation of LC3 interacting region (LIR) on ATGL can completely block the transport of ATGL to LDs, while the enhanced localization of LC3-II, the classical markers of autophagic vesicles, on LDs can activate the occurrence of lipophagy (Martinez-Lopez et al., 2016). In a recent study, it has been found that ATGL-driven lipolysis targets large LDs to reduces the size of existing LDs in hepatocytes, which play a role upstream of lipophagy (Figure 1B; Schott et al., 2019). Significantly, lipolysis mediated reduction in LDs volume to reach a size threshold for autophagosome engulfment and the fusion with lysosomes during lipophagy. In addition, free fatty acids released during ATGL mediated lipolysis can upregulate lipophagy through the deacetylase sirtuin 1 (SIRT1) dependent transcriptional regulation (Figure 1B; Sathyanarayan et al., 2017). Together, these studies all demonstrate a significant interdependence between lipolysis and lipophagy.

#### Molecular Events Occurring With Macrolipophagy

**Figure 2:**
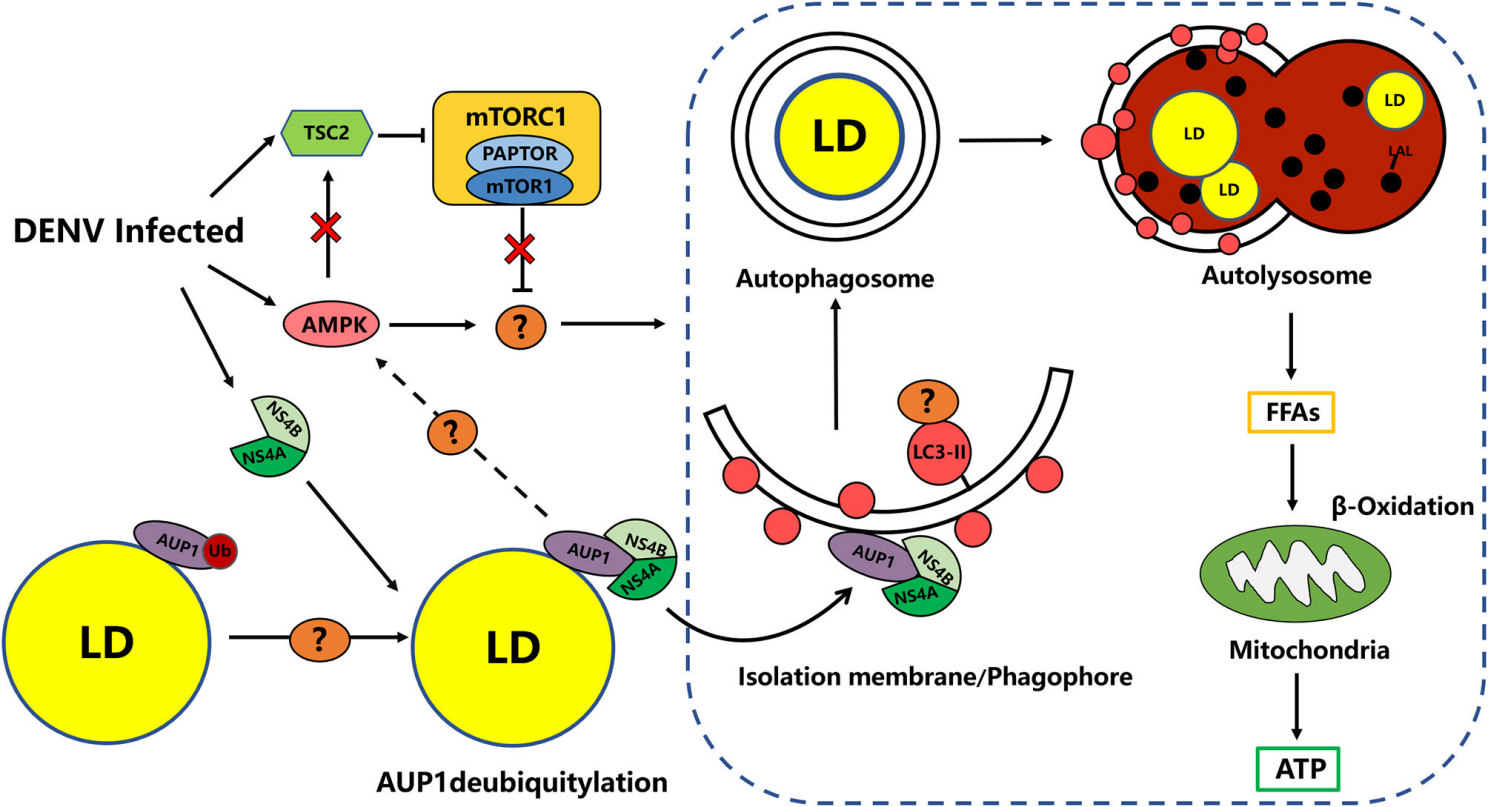
DENV-induced Lipophagy depletes LDs to provides energy for virus replication. DENV infection activates and requires AMPK signaling as well as AMPK-independent inhibition of mTORC1 activity to target unknown candidate to induce proviral lipophagy. DENV infection induces the deubiquitylation of AUP1 by an unknown mechanism. DENV NS4A/4B binds the deubiquitylated AUP1 and relocalizes it to the autophagy initiation. AUP1 relocalization may trigger lipophagy through the AMPK/mTOR pathway. Phagophores may be targeted to LDs through unknown selective autophagy receptors. DENV upregulate lipophagy dependent on the AUP1 lysophospholipid acyltransferase activity to release FFAs from LDs, for subsequent catabolic processing via β-oxidation (Lin et al., 2017).

Fundamentally, macrolipophagy, as a form of selective autophagy, refers to the canonical autophagosome-mediated pathway of budding off and sequestering LDs (or portions) for subsequent delivery to the autolysosome in which the LDs are degraded, the molecular basis of which is the attachment of tagged cargo to the autophagosome via adaptor proteins (Khaminets et al., 2016; Schulze et al., 2017; Takats et al., 2019). Based on the current study we found that there are multiple possible mechanisms for recruiting autophagosomal membranes to LDs. It is known that during selective autophagy, different autophagy receptors, such as CALCOCO2/NDP52, SQSTM1/p62, NBR1, and others, interact with LC3-II, a classical marker of autophagic membranes, through LC3-interacting region (LIR) motifs. In addition to LDs, almost all intracellular organelles have unique membrane-bound receptors involved in the autophagic machinery. For example, NDP52 and optineurin were identified to be involved in the selective process of mitophagy (Schulze et al., 2017). A previous study revealed that rapamycin-driven autophagy promotes p62 localization on LDs by interaction with PLIN 2 (Figure 1C). Furthermore, the recruitment of p62 to LDs effectively decreases lipid accumulation in cardiomyocytes, which suggest that p62 may function as an autophagosomal membrane-bound receptor to direct autophagosomes to LDs and accelerate lipid degradation (Lam et al., 2016). In addition, protein modification by polyubiquitination may serve as a signal to promote lipophagy. Regarding LDs ubiquitination, it has previously been suggested that the interaction between the ancient ubiquitous protein 1 (AUP1) and the E2 ubiquitin conjugase G2 (UBE2G2) at the LDs surface may modulate the onset of lipophagy (Spandl et al., 2011; Stevanovic and Thiele, 2013). Nevertheless, recent studies subvert previous observations that AUP1 deubiquitination is closely linked to the triggering of lipophagy rather than its ubiquitinated form (Figure 2; Zhang J. et al., 2018). Therefore, in contrast to selective autophagy targeting other organelles, the evidence for whether ubiquitination or classical selective autophagy adaptor proteins serving a major role in lipophagy is an overall paucity.

According to previous findings, the Rab GTPase family may also be involved in this process (Schroeder et al., 2015; Li Z. et al., 2016). It appears that Rab7 mediates the recruitment of multivesicular bodies and lysosomes to LDs during lipophagy (Schroeder et al., 2015). In the meanwhile, Rab10 also localizes to LDs and autophagosomes in a Rab7 dependent manner, which is essential for the migration of LC3-positive autophagic membranes to the LDs surface. In this process, it forms a complex with the adapter protein EHBP1 and EHD2, which could mechanically drive the extension of the autophagosome membrane around the LDs for engulfment (Figure 1C; Li Z. et al., 2016; Zhang X. et al., 2018). In addition, other Rab GTPase, such as Rab18, an obligate type of rabgtpase present only on LDs, as well as Rab25 and Rab32 have been found to localize to LDs and may be involved in the process of lipophagy, while the specific molecular mechanism remains unclear (Ozeki et al., 2005; Li Q. et al., 2016; Chen et al., 2017). In this context, there are many roles played by Rab GTPases associated with LDs remain undefined.

#### Degradation of LDs Proteins by CMA Promotes Lipolysis

The PLIN family proteins have been shown to coat LDs and protect TG from degradation by cytoplasmic lipases. As shown in Figure 1A, when the organism is under starvation, PLIN 2 and PLIN 3 can be degraded by CMA, which increases the access of lipases to neutral lipids and basal lipolysis (Kaushik and Cuervo, 2015, 2018). In CMA, the target protein with a KFERQ motif is recognized by the heat shock cognate protein (Hsc70) complex and shuttled to the lysosomal surface, where the unfolded substrate protein is translocated into lysosomal lumen and degraded via the binding of Hsc70 and lysosome-associated membrane protein 2A (LAMP2A) (Figure 1A; Yang Q. et al., 2019). Lysosomes with CMA activity were initially extracted from starved rat livers, and reduced expression of PLIN 2 and PLIN 3 proteins in lysosomal fractions was detected, confirming that PLIN 2 and PLIN 3 are substrates of CMA. Readily in oleic acid treated mouse fibroblasts, Hsc70, which recognizes the pentapeptide KFERQ motif, was found to interact with PLIN 2/3 (Kaushik and Cuervo, 2015). A follow-up study revealed that PLIN 2 (may be including PLIN 3), the substrate of CMA, forms p-PLIN 2 by phosphorylation (P) through AMP-activated protein kinase (AMPK) and translocation from the LD surface to lysosomes, where it promotes the recruitment of a neutral lipid lipase (ATGL) and macrolipophagy machinery components to the LD (Kaushik and Cuervo, 2016). In an interesting study, brief kiss-and-run events as well as prolonged interactions between LDs and lysosomes were observed. These organelles contact sites make PLIN 2 and PLIN 3 translocate from LDs to lysosomes. However, the mutation of CMA-targeting motif of PLIN 2 reduced LD-lysosome contacts, suggesting that such LD-lysosome contacts may be relevant for PLINs, which may provide initial clues to the occurrence mechanism of microlipophagy. However, there is currently a lack of evidence demonstrating microlipophagy in mammalian cells. In addition, the previously mentioned small GTPase Rab7 is also involved in the formation of LD-lysosome contacts and facilitates PLIN 2 transfer from LDs to lysosomes and lipophagy process (Figure 1D; Schroeder et al., 2015). Taken together, CMA can target LDs to mediate the degradation of PLIN 2 and PLIN 3 and play unexpected indirect roles in lipolysis and macrolipophagy genesis. Furthermore, it remains uncertain whether other LDs surface proteins act as degradation substrates for CMA and needs to be explored further.

## Regulation of Lipophagy in *Flaviviridae* Infection

As an important sub process of autophagy, lipophagy not only regulates cellular lipid metabolism, but is also closely related to infection with a variety of pathogenic microorganisms (Schulze and McNiven, 2019; Yang L. et al., 2019). Multiple previous studies have indicated that a variety of pathogenic microorganisms, particularly *Flaviviridae* virus (HCV, DENV, CSFV, etc.) can exploit host resources, evade host immunity mechanisms and hijack autophagy to facilitate its own replication, which may also be a potential mechanism by which they utilize LDs (Heaton and Randall, 2010; Su et al., 2011; Pei et al., 2014, 2016; Liang et al., 2016; Fan et al., 2020). It has been suggested that the degradation of TG within LDs into FFAs is the sole carbon source for *Mycobacterium tuberculosis* (*M. tb*) (Daniel et al., 2011). Inhibition of lipophagy profoundly reduced the fatty acid β-oxidation capacity of the host cells and significantly decreased the survival of *M. tb bacilli* (Barisch et al., 2015; Barisch and Soldati, 2017) In addition to bacteria, it is also crucial for the replication and infection of some viruses, as briefly illustrated here using DENV, ZIKV, and hepatitis virus as examples. Currently, the relationship between lipophagy and viral infections has already been explored (to a greater or lesser extent) in the context of DENV, ZIKV, JEV, and HCV, which we will briefly illustrate here.

### Lipophagy and the *Flaviviruses* Represented by DENV

Several studies have reported that DENV induces proviral autophagy (Lee et al., 2013; Metz et al., 2015). Consistent with these findings, studies in several cell types have shown that DENV infection increases autophagosome accumulation and blocking autophagy is able to strongly reduce viral replication (Heaton and Randall, 2010; Mateo et al., 2013; Jordan and Randall, 2017). In a follow-up study, DENV can induce a form of proviral selective autophagy targeting LDs, called lipophagy, which stimulates lipid metabolism. This study found that DENV infection of hepatocytes quickly increases the number of autophagosomes associated with LDs and transfers them to lysosomes for degradation (Jordan and Randall, 2017). Interestingly, the induction of lipophagy by DENV infection significantly reduced the amount of LDs and TG in the cells, while the cholesterol levels are only modestly perturbed, which may reflect a selective processing of TG. Alternatively, the reduction in cholesterols may be offset by enhanced cholesterol synthesis and transport at sites of DENV replication (Rothwell et al., 2009; Heaton and Randall, 2010). Randall et al. found that the DENV NS4A protein was translocated into the cytoplasm to form a membrane replication complex, and in parallel with the induced lipophagy to deplete LDs and TG for FFAs release, where finally ATP generated by β-oxidation in mitochondria is required for DENV RNA replication (Heaton and Randall, 2010). Consistent with this model, the investigators found a reduction in LDs volume during DENV infection by electron microscopy, and the defect in DENV replication after inhibition of autophagy can be compensated by exogenous fatty acids (Randall, 2018). In addition, a recent study further showed that the smaller LDs are preferentially targeted for autophagy rather than cytosolic lipolysis (Schott et al., 2019).

Lipophagy is an understudied but important contributor to lipid degradation and signaling pathways that control lipophagy remain unclear. Similarly, the mechanism of how DENV specifically triggers lipophagy rather than basal autophagy during viral infection is not fully understood. Based on this, it was found in a follow-up study that DENV infection activated the central metabolic regulator AMPK, whereas silencing or pharmacological inhibition of AMPK activity reduced DENV replication and induction of lipophagy. Correspondingly, the activity of mammalian target of rapamycin complex 1 (mTORC1) was reduced and inversely correlated with lipophagy induction in DENV infected-cells. Although AMPK normally stimulates tuberous sclerosis complex 2 (TSC2)-dependent inactivation of mTORC1 signaling (Jacobs et al., 2017), mTORC1 inactivation occurs independently of AMPK activation during DENV infection, and the researchers suggest that it may has additional candidates that regulate lipophagy, such as ULK1/2 and Vps34-Beclin complexes (Figure 2). Therefore, the investigators proposed that DENV stimulates and requires AMPK signaling as well as AMPK-independent inhibition of mTORC1 activity to induce proviral lipophagy in DENV-infected cells, which is required for the induction of lipophagy and the robust DENV replication, but not basal autophagy (Figure 2; Jordan and Randall, 2017; Xiao and Cai, 2020).

Actually, the induction of lipophagy is generally poorly understood, but a study has provided important clues to the underlying molecular mechanism of DENV-induced lipophagy. AUP1, a multifunctional type III membrane protein on LDs, was previously implicated in regulating LDs metabolism and ER-associated degradation (ERAD), and recently it was found to play a relevant role in lipophagy induced by DENV and other *flaviviruses* such as ZIKV and WNV (Klemm et al., 2011). In this process, the researchers demonstrated that DENV NS4A/B binds to AUP1 and promotes its translocation from LDs to autophagosomes to drive induction of lipophagy (Figure 2; Randall, 2018; Zhang J. et al., 2018). Importantly, AUP1 is required for DENV induced-lipophagy, while AUP1-deficient cells were still able to induce autophagy in general, suggesting that AUP1 may be a specific inducer of DENV-promoted lipophagy. At present, the specific mechanism by which AUP1 triggers lipophagy has not been fully elucidated, and current evidence suggests a close relationship with its acyltransferase activity and modification by ubiquitination. Interestingly, it is deubiquitinated-AUP1 that interacts with NS4A, while the ubiquitinated-AUP1 instead disrupts its association with NS4A and inhibits the trigger of DENV-induced lipophagy (Zhang J. et al., 2018). Moreover, deletion of AUP1 arrests DENV-induced lipophagy and impairs the production of infectious DENV and ZIKV progeny viruses in the human HepG2 cell line (Zhang J. et al., 2018). In the latest study, researchers found that depletion of LDs and increased production of DENV were promoted by phosphatase and tensin homolog deleted on chromosome 10 (PTEN) and its mutant Y138L mediated autophagy. In this process, PTEN lipid phosphatase activity can decrease the area and number of cellular LDs by enhancing autophagy via Akt/FoxO1/Maf1 signaling, which ensure the sufficient supply of lipids for DENV replication and assembly (Liu et al., 2020). In summary, these findings provide mechanistic insight into the role of LDs utilization during DENV infection.

Similarly, the NS4A and NS4B proteins of ZIKV have also been found to induce lipophagy and thereby inhibit neurogenesis in human embryonic neural stem cells. The occurrence of ZIKV-induced lipophagy was similarly demonstrated in a recent study on the reorganization of organelles upon ZIKV virus infection of different cell lines, which found that the number and volume of LDs were reduced in ZIKV-infected cells, and this virus might induce LDs biogenesis, which stimulate the initial viral replication, and then trigger lipophagy to reduce LDs and release FFAs from these lipid structures (García et al., 2020). Meanwhile, the Japanese encephalitis virus (JEV) infection, analogous to other *flaviviruses* (DENV, WNV and ZIKV), decreased the number of LDs per cell indicating a link between lipid metabolism and virus replication (Sarkar et al., 2020). Based on these mentioned above, lipophagy, which degrades intracellular lipids and provides energy for viruses, is a key process in *flaviviruses* replication. Nevertheless, the investigation of mechanisms underlying lipophagy induction of these viruses are still poorly understood.

### Lipophagy and HCV

Early studies suggested that lipid and/or sterol biosynthesis and modification are essential for replication of some picornaviruses and (+) RNA viruses (Cherry et al., 2006), Such as hepatitis viruses are not only able to orchestrate autophagy to modulate the lipids and evade immune surveillance, but also to disrupt host lipid metabolic homeostasis to guarantee their own stable survival. The life cycle of hepatitis viruses is closely related to the host lipid landscape, especially HCV (Syed et al., 2010; Del and Romero-Gomez, 2015).

On the HCV nature, Studies have reported that although HCV does not directly modulate lipid changes, it can indirectly modulate lipid levels by inhibiting HCV-induced alterations in lipid metabolism via autophagy, and it may contribute to the development of hepatic steatosis when autophagy is disrupted. That is, autophagy levels inversely correlate with HCV steatosis (Vescovo et al., 2012; Bassendine et al., 2013). As mentioned earlier, with the secretion of very low-density lipoprotein (VLDL), HCV LVPs formed via NS5A binding to ApoE or ApoA are targeted for transport to hepatocytes. Moreover, the expression of HCV core protein, a key modulator of viral and cellular gene expression, and NS5A protein would cause LDs aggregation, inhibit TG transport, and lead to fat accumulation (Mancone et al., 2011). These studies directly imply the possibility that the roles played by LDs in HCV are multiple. LDs serve as a desirable platform for HCV assemble, and inducing lipophagy can sustain the high level of ATP required for viral replication (Bose and Ray, 2014; Meyers et al., 2016). However, there are few reports on how HCV activates lipophagy for lipolysis, and a recent study found that LDs-associated α/β hydrolase domain containing protein 5 (ABHD5), also known as comparative gene identification-58 (CGI-58), plays an important role. ABHD5 is a highly conserved regulator of ATGL-mediated lipolysis, which was recently proved to be critical for HCV assembly and release, and it can mobilize LDs-associated lipids likely via the regulation of ATGL expression and lipase activity (Bley et al., 2020; Vieyres et al., 2020). This study may provide a reference for HCV to activate lipophagy through ATGL. In addition, in an earlier study, researchers found that HCV NS4B binding with Rab5 and vps34 can activate autophagy, while NS5B binding with ATG5 of autophagy is involved in the extension of autophagy membrane (Su et al., 2011), from which we may speculate that the activation of HCV-induced lipophagy may be closely related to NS4B and NS5B. We hypothesized that HCV may affect lipophagy in the host by modulating autophagic function. On one hand, HCV impedes the host transcription factors expression, especially genes that control lipolysis, and the activation of lipophagy *in vivo* leads to lipid aggregation, on the other, lipophagy is utilized to the LDs degradation to release free lipids to provide energy for the viral RNA replication, assembly, and transport (Carmona-Gutierrez et al., 2015).

## Discussion

Over the past decade, our knowledge of the importance of viral remodeling of cellular metabolism has increased dramatically. Although many examples have been well-documented, there is still relatively little understanding of the detailed mechanisms of how these metabolic alterations are promoted. LDs are the crux of replication assembly of *flaviviruses* and even *Flaviviridae*, and autophagy/lipophagy plays a pivotal role in the *Flaviviridae* infection process by regulating LDs metabolism. Its common primary model is that viruses upregulate lipophagy to release FFAs from LDs, for subsequent catabolic processing via β-oxidation, providing *Flaviviridae* with favorable conditions similar to the lipid microenvironment for infection and replication. Therefore, in-depth mining of the interaction of lipophagy with pathogenic infection and the mechanisms of its induction is of great significance to reveal the pathogenic mechanisms of viral infection. Moreover, the screening of new antiviral drugs that target host LDs or modulate LDs metabolic processes (e.g., lipophagy) has the potential to be new observational approach of metabolic disorders as well as infectious diseases.

In view of the fact that lipid metabolism is one of the major cellular pathways that can be intervened by drugs, the idea of repositioning drugs targeting lipid metabolism as antiviral drug candidates is gaining progressive support (Martín-Acebes et al., 2019). For instance, upon HCV infection, core proteins are able to induce steatosis in the liver, an effect that requires the participation of TIP47, and the knockdown of TIP47 expression resulted in a corresponding decrease in core protein expression, which may serve as an effective way to attenuate the pathological response induced after HCV infection. Indeed, the abundance of LDs can be controlled by Subtilisin Kexin Isozyme-1 (SKI-1)/Site-1 Protease (S1P) inhibitors like PF-429242, which may aid in the development of host-targeting agents (HTAs) for *flavivirus* control (Hyrina et al., 2017). Such HTAs could be broadly therapeutic against not only *Flaviviridae*, but also other pathogens hijacked lipids (Roingeard and Melo, 2017; Cloherty et al., 2020). In consideration of the current COVID-19 pandemic caused by SARS-CoV-2 (severe acute respiratory syndrome coronavirus 2), a recent preliminary data has showed that inhibitors of lipid metabolism and autophagy can inhibit SARS-CoV-2 replication *in vitro* (Silvas et al., 2020). HTAs offer an intriguing chapter for novel antiviral strategies since they have a pan-genotypic antiviral activity and a high genetic barrier to drug-resistance.

Targeting of LDs by lipophagy has an important role for the regulation of cellular homeostasis. Today, there is ample evidence to prove that lipophagy is also very closely related to the infection of a variety of pathogenic microorganisms. Although there have been major breakthroughs in the field of lipophagy research, it is still in its infancy, and research on the factors and specific pathways involved in this complex process is still ongoing. Taking the molecular mechanism of lipophagy induction by DENV as an example, at present we have gained more insight into how DENV induces lipophagy. However, a series of questions remain to be elucidated in this process (Figure 2). By what deubiquitinase does DENV infection trigger AUP1 deubiquitination and subsequently enable NS4A/B to associate with it? How does NS4A/B promote the relocalization of AUP1 to autophagosomes and then trigger autophagy? Is the engagement of relevant selective autophagy adaptors such as p62, among others, required? What signaling pathways might be involved in AUP1-triggered lipophagy? What is the hallmark of LDs as autophagosome substrates? Is it alike with CMA? AUP1-induced lipophagy is able to affect viral assembly, and what is the specific role of lipophagy in viral assembly, in addition to providing energy? The specific molecular mechanism remains unclear and requires further investigation. Overall, mechanistic studies on the occurrence of DENV-induced lipophagy provide a great deal of insight into the mechanism of lipophagy in other *Flaviviridae* infections.

Regarding the process by which viral infection induces lipophagy, there are currently only sporadic clues, apart from the Rabs, the cross-talk between lipases and the core lipophagy machinery, mentioned in this review that induce and regulate the occurrence of lipophagy, many other factors are involved such as the transcription factor EB (TFEB) and Forkhead box protein O1 (FoxO1), calcium signaling transduction, small molecules including ethanol, etc. How these cues relate to each other, and what are the triggering molecular mechanisms specific to lipophagy are unclear. To this end, the dynamic relationship of lipophagy and viral infection remains to be further explored.

## Author Contributions

KW: writing—original draft preparation. SF, LZ, and FZ: writing—review and editing. MZ, HY, and JC: conceptualization, supervision, and project administration. All authors contributed to the conception and design of the work. All authors have read and agreed to the published version of the manuscript.

## Conflict of Interest

The authors declare that the research was conducted in the absence of any commercial or financial relationships that could be construed as a potential conflict of interest.

## Abbreviations

HCV: hepatitis C virus
DENV: dengue virus
DSS: dengue shock syndrome
ZIKV: Zika virus
BVDV: bovine viral diarrhea virus
CSFV: classical swine fever virus
LDs: lipid droplets
TG: triacylglycerol
CE: cholesteryl esters
ER: endoplasmic reticulum
PLIN A: Perilipin A
ADRP/PLIN2: adipose differentiation related protein
TIP47/PLIN3: 47-kDa tail interacting protein
ATGL: adipose triglyceride lipase
HSL: hormone- sensitive lipase
nCEH: neutral cholesterol ester hydrolase
LVP: lipoviroparticle
Rabs: Ras-related GTP-binding proteins
SNAREs: soluble NSF attachment protein receptors
EV71: enterovirus71, RV, rotaviruses
CAMP: cathelicidin
MGL: monoacylglycerol lipase
LAL: lysosomal acid lipase
FFAs: free fatty acids
3MA: 3-methyladenine
CMA: chaperone mediated autophagy
CALCOCO2/NDP52: calcium binding and coiled-coil domain 2
NBR1: SQSTM1/p62, sequestosome 1
NBR1: autophagy cargo receptor
P: phosphorylation
AMPK: AMP-activated protein kinase
AUP1: ancient ubiquitous protein 1
UBE2G2: E2 ubiquitin conjugase G2
EHBP1: EH domain-binding protein 1
EHD2: membrane-modified adenosine triphosphatase (ATPase) EH domain containing 2
Hsc70: heat shock cognate protein complex 70
LAMP2A: lysosome-associated membrane protein 2A
TSC2: tuberous sclerosis complex 2
mTORC1: mammalian target of rapamycin complex 1
ERAD: ER-associated degradation
PTEN: phosphatase and tensin homolog deleted on chromosome 10
JEV: Japanese encephalitis virus
VLDL: very low-density lipoprotein
ABHD5: associated α/β hydrolase domain containing protein 5
CGI-58: comparative gene identification-58
SKI-1: Subtilisin Kexin Isozyme-1
S1P: Site-1 Protease
HTAs: host-targeting agents
SARS-CoV-2: severe acute respiratory syndrome coronavirus 2
TFEB: transcription factor EB
FoxO1: Forkhead box protein O1.
